# Using administrative register data for adjusting non-response bias in the finnish gambling harms survey

**DOI:** 10.1186/s12889-025-23016-4

**Published:** 2025-05-16

**Authors:** Jukka Kontto, Hanna Tolonen, Anne H. Salonen

**Affiliations:** 1 Finnish Institute for Health and Welfare, Department of Public Health, P.O. Box 30, Helsinki, FI-00271 Finland; 2 Finnish Institute for Health and Welfare, Helsinki, Finland; 3 Faculty of Health Sciences, University of Eastern Finland, Kuopio, Finland; 4 Finnish Institute for Health and Welfare, Department of Healthcare and Social Welfare, Helsinki, Finland

**Keywords:** Response rate, Gambling, Non-response bias, Population survey, Register-based data, Socio-demographics

## Abstract

**Background:**

Low response rates are an increasing problem in population-based gambling surveys. Selective non-response may cause biased findings. Supporting information from administrative registers, whenever available for non-respondents can be utilized to estimate the effect of non-response to the gambling-related outcomes. The aim of this study is to evaluate the effect of non-response to the prevalences of two gambling measures: gambling participation and problem gambling.

**Methods:**

Population-based Finnish Gambling Harms mixed-mode (online and postal) Survey 2016 was conducted among 18-year-olds or older in three geographical regions in Finland (response rate 36.2%). Weighted prevalences of gambling measures were calculated exploiting the respondents’ data (*n* = 7,153). The study sample (*N* = 19,741) was individually linked to socio-demographic data from Statistics Finland to obtain information on both respondents and non-respondents. Multiple imputation was utilized to calculate the adjusted prevalences of gambling measures by register-based variables: sex, age, residential area, family structure, household equivalised disposable income, highest education degree, employment status, and native language. Crude prevalences were compared against weighted and non-response adjusted prevalences.

**Results:**

For gambling participation, there was no difference between the crude (81.9% [95% CI 81.0–82.8%]) and the weighted (83.2% [95% CI 82.3–84.0%]) prevalences (p-value 0.09), or between the crude and the non-response adjusted (82.3% [95% CI 81.6–83.0%]) prevalences (p-value 0.49). However, the non-response adjusted (2.8% [95% CI 2.4–3.3%]) prevalence of problem gambling was higher compared to the crude (1.9% [95% CI 1.6–2.3%]) prevalence (p-value 0.002), while there was no difference between the crude and the weighted (2.2% [95% CI 1.9–2.7%]) prevalences (p-value 0.26).

**Conclusions:**

Non-response had an effect of problem gambling prevalence in a Finnish Gambling Harms Survey 2016. The presence of non-response bias should be checked when analysing population surveys. Using administrative register data enables unique opportunities to increase the reliability of the results and to adjust the estimates for non-response.

**Trial registration:**

Clinical trial number: not applicable.

**Supplementary Information:**

The online version contains supplementary material available at 10.1186/s12889-025-23016-4.

## Background

Trends in Finnish gambling participation and problem gambling has been monitored using population-based studies [1], as in many other Western countries [2–5]. Random sampling methods are widely accepted as the best foundation for representative data. The quality of the measurement, systematic and random population coverage-related errors as well as representativeness of the target population has an impact on interpretation of survey results. However, low response rates are an increasing problem with population-based gambling surveys and vary depending on the data collection mode/methods and time [6]. The response rates of all types of surveys in general have continued to decrease internationally in the last ten years, and often already 30–40% response rate is considered good [7, 8]. At the same time, combinations of online and postal surveys have become more popular, even though challenges with their response rates are known. Understanding non-response and finding methods to increase representativeness of the data are essential, while these actions also provide information which can be exploited for reaching higher response rates. Therefore, the impact of non-response is well worth assessing [6].

Non-response, if selective, may cause biased findings. Supporting information from administrative registers, whenever available for non-respondents, can be used to define the profile of non-respondents. In gambling surveys, the association of non-response and socio-demographic factors, for instance sex, age, family structure, socio-economic status, residential area, ethnic background, has been studied with the results being somewhat inconsistent.

Non-respondents are typically more often men [6, 9–16]. On the other hand, based on the Finnish Gambling 2015 and 2019 studies, the response rate was higher among men compared with women [1, 17]. Further analysis with the 2015 data indicated that the sex difference disappeared when adjusting for net income [18]. The Finnish Gambling 2023 study had higher response rate for women compared to men [19].

Younger age is often associated with lower response rate [1, 12–16, 19, 20]. Furthermore, if there was no upper age limit for a study, the highest age group (85-year-olds or older) also had lower response rate [6]. In Sweden, however, there has been inconsistency in results. In 2015 study, the youngest (16–19-year-olds) were most active to participate, and 20–39-year-olds had the lowest response rate [9], while in most recent Swedish study conducted in 2021, 20–24-year-olds had the lowest response rate, while 70–84-year-olds had the highest [11].

The association between family structure and non-response is inconsistent. Non-respondents were more likely to live in large families or adult households [16]. Also in the most recent Swedish study, living in family without children was associated with higher response rate compared to living in family with children or being single with or without children [21]. In a Danish population study, the response rate was especially low among unmarried persons [6].

In Great Britain, having a responsible adult or household reference person who has been engaged in routine occupation have been associated with non-response [16]. Low socio-economic status, such as low education, low income, unemployment and/or receiving social welfare, has been linked with lower response rate [6, 18, 21]. However, Kontto et al. noticed that when net income was added to the non-response model with the above-mentioned Finnish data, the link between unemployed status (vs. self-employed) and non-response became non-significant, while the non-response was more prevalent among the lowest quintile of net income [18]. This may cause bias while studying gambling behaviour of socio-economically vulnerable individuals.

The impact of the residential area is not clear. In Sweden, the response rates were similar between those living in urban and non-urban areas [21]. On the other hand, some studies have reported differences between response rates by area [14, 16]. In Denmark and Iceland, those living in capital area participated significantly less often than those living in other areas [6, 13].

The response rate was especially low among persons with a different ethnic background than Danish which might be explained by the fact that the questionnaire was only available in Danish [6]. Also, people born outside Nordic countries had a low response rate [21].

The profile of non-response may cause bias in the prevalence of gambling measures, for instance gambling participation and problem gambling. In recent Nordic gambling studies, the prevalence of gambling participation was higher among men, older age groups, higher socio-economic groups, and individuals living in rural areas [11, 13, 14, 19]. However, the same socio-demographic factors which are associated with problem gambling are also associated with non-response [11, 13, 14, 19, 20, 22–24]. This may cause more bias to the prevalence estimates of problem gambling compared to gambling participation.

A method widely used for lowering the bias caused by non-response is weighting [25]. Auxiliary information, for instance demographic characteristics, on the population is exploited to ensure that the results are representative of the population. However, the number of variables used for weighting is often quite limited, leaving out characteristics, for instance socio-demographic factors, which may have an association with non-response.

Information about non-response can be used to estimate the effect of non-response to the study outcomes by obtaining additional auxiliary information through record linkage to administrative registers. This individual-level data on both respondents and non-respondents provides an opportunity to use sophisticated methods for adjusting bias caused by non-response, such as multiple imputation (MI). To our knowledge, these methods have not been used so far with gambling-related outcomes and our study aims to fill this gap in knowledge. Therefore, the aim of this study is to evaluate the effect of non-response to the prevalences of gambling participation and problem gambling in the Finnish Gambling Harms Survey 2016 [26]. The crude prevalences are compered to non-response adjusted prevalences calculated using MI. In comparison, crude prevalences are compared to weighted prevalences.

## Methods

### Participants and procedure

The Finnish Institute for Health and Welfare was responsible for design and analysis of data of the population-based Finnish Gambling Harms Survey 2016, while Statistics Finland was responsible for the data collection. Originally, the aim of this longitudinal study was to evaluate gambling, gambling-related harm, and opinions on gambling marketing linked with the reform of the Finnish gambling monopoly. Finnish Gambling Harms Survey 2016 was conducted in three selected geographical regions (Uusimaa, Pirkanmaa, and Kymenlaakso) covering for around 42% of the total Finnish population using mixed-mode survey [26, 27]. The sample consisted of six age-region strata with two age groups (18–24-year-olds, and 25-year-olds or older) from each of three regions. The study sample was selected by strata using systematic random sampling from the population frame sorted by domicile code from the national population information system based on the following criteria: (1) 18-year-old or older, (2) the ability to understand Finnish or Swedish, and (3) excluding institutionalized persons, such as prisoners, mental health patients and the infirm. 18–24-year-olds were oversampled due to fact that problem gambling being most prevalent is that age group: 15% of 18–24-year-olds were sampled for the survey while they represent only 10% of the population [26].

The data collection mode included online and postal surveys, which were available in both Finnish and Swedish. The invitation letter for the invitees was sent to their home address retrieved from the population information system. Both the invitation letter and the first reminder included a link to the web questionnaire and a personal participation code. In this letter, information about the upcoming option to answer to the paper questionnaire was provided. The next two reminder letters also included the paper questionnaire and a prepaid return envelope. The data collection was conducted between January and March 2017.

**Fig. 1:**
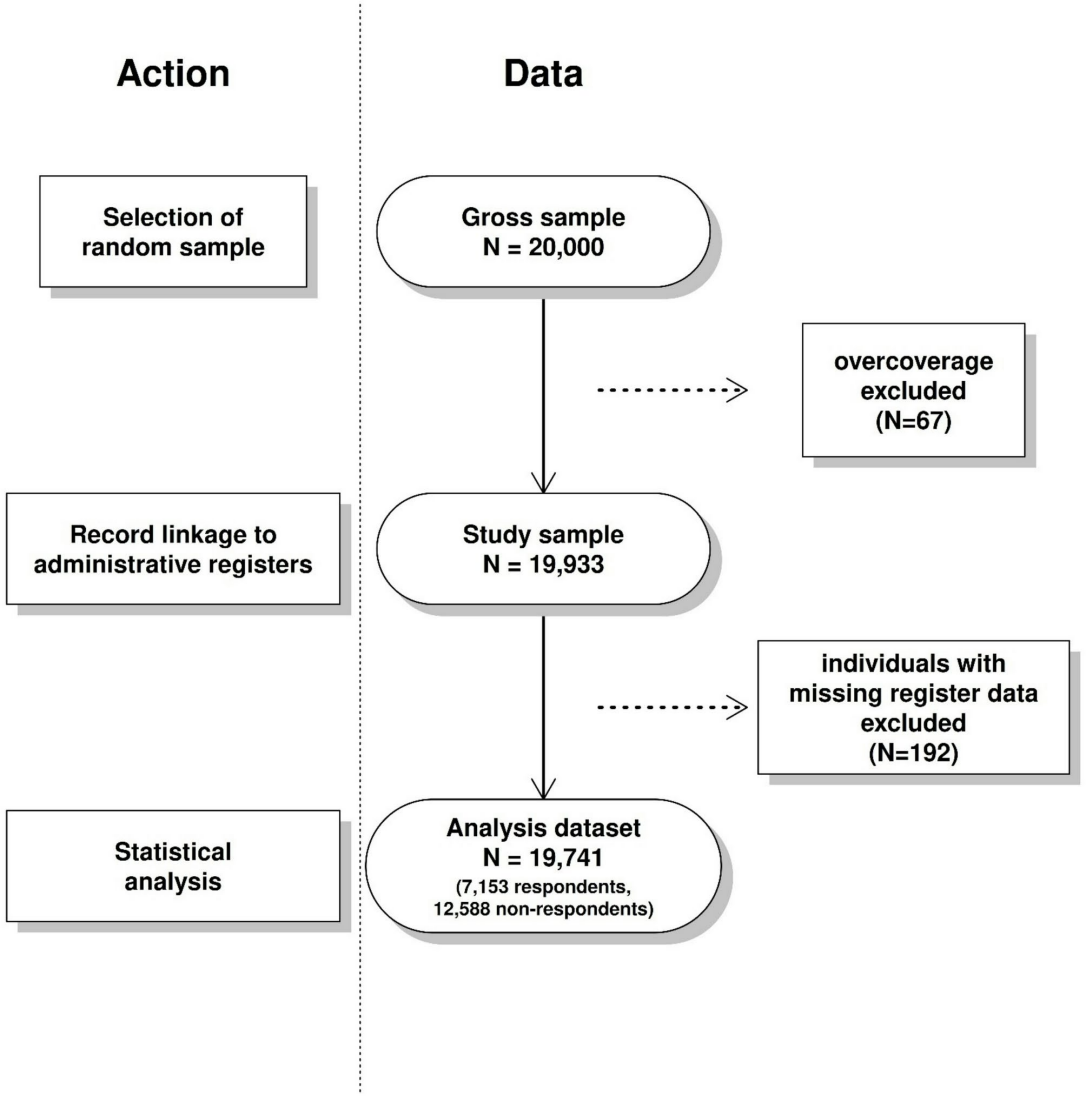
Flowchart of the exclusion of sample subjects from the gross sample to the analysis dataset

After excluding overcoverage (*N* = 67), the study sample size was 19,933 persons. Ultimately, 7,186 participated in the study. Of the respondents, 71% (*N* = 5,084) participated using the online survey while 29% (*N* = 2,102) completed the postal survey. The study sample was linked to administrative socio-demographic data from Statistics Finland through personal identification code issued to every Finnish citizen to obtain information on both respondents and non-respondents. 192 individuals were excluded from the study sample due to missing household information with the reasons for missingness including living abroad, homelessness, living in uncommon or unusual large household, and the place of residence being unknown. The final analysis data consisted of 7,153 respondents and 12,588 non-respondents (*N* = 19,741) with a response rate of 36.2% (Fig. 1).

### Measures

#### Socio-demographic variables

The administrative socio-demographic data from Statistics Finland for all individuals in the study sample was obtained for following variables (categories in parenthesis): sex (men, women), age (continuous), number of family members (continuous), number of under 18-year-old family members (continuous), number of household members (continuous), household’s disposable income (continuous), highest education degree (primary, secondary, and tertiary), employment status (unemployed, entrepreneur, white-collar, blue-collar, student, retired, and other or missing), residential area (rural, semi-urban, and urban) and native language (Finnish, Swedish, and other).

Household equivalised disposable income (HEDI) was derived by dividing the household’s disposable income by its equivalent size. The household’s equivalent size was calculated as the sum of weights in a household. A weight was assigned to all household members using a following rule: 1.0 to the first adult; 0.5 to the second and each subsequent 18-year-old or older family member, and to all non-family household members regardless of age; 0.3 to each under 18-year-old family member. There was no information available concerning the age of non-family household members. The rule was a modification to the definition by Eurostat Statistics Explained definition [28] where the cut-off age is 14 years and ages of all household members is known. HEDI was categorized into tertiles. Family structure was derived from number of family members and number of under 18-year-old family members resulting following categories: living alone, family with children (under 18-year-olds), and family with only adults (18-year-olds or older). Age was categorized into categories 18–24, 25–34, 35–44, 45–54, 55–64, 65–74, 75–84, and 85 + years, since the response rate decreased among respondents older than 85 years (Supplementary Fig. 1).

#### Gambling measures

Gambling participation and problem gambling were derived only for the respondents. In the questionnaire the time frame for both gambling participation and problem gambling was past year, i.e. the calendar year 2016. Gambling participation variable has two categories (0 = ‘not a past-year gambler’ and 1 = ‘past-year gambler’). A respondent was classified as a past-year gambler if he/she had gambled at least one game type (out of 18 listed game types) during the previous year, (i.e. year 2016) and non-past-year gambler otherwise. Gambling refers to playing games for example lottery games, slot machines, scratch cards, sports and horse games, betting games which are also available online.

Problem gambling variable has two categories (0 = no problem gambling’ and 1 = ‘problem gambling’). Past-year gambling severity was evaluated using the 14-item Problem and Pathological Gambling Measure (PPGM) [29]. The PPGM includes three categories: Problems (7 questions), Impaired Control (4 questions), and Other Issues (3 questions). PPGM scores were divided into four categories: recreational gambling, at-risk gambling, problem gambling and pathological gambling. In our study, PPGM questions we asked only from those, who had gambled at least once a month in 2016, and those who had gambled less frequently than monthly were defined recreational gamblers. Furthermore, an additional category was created for those respondents who did not gamble at all. A binary problem gambling variable was derived by combining problem gambling and pathological gambling to indicate problem gambling with all the remaining categories indicating non-problem gambling.

### Statistical analysis

Socio-demographic factors sex, age, residential area, family structure, HEDI, highest education degree, employment status, and native language were included in the analyses. Crude and weighted prevalences were calculated using complete case analysis, i.e. exploiting only the respondents with non-missing gambling measures.

For weighted prevalences, Statistics Finland provided the survey weights for each respondent after the following process: First, the design weights were calculated using the six age-region strata. Then, design weights were calibrated to match the population distributions of sex, age, education degree, region, and urban–rural classification using SAS macro Calmar 2 for correcting the possible bias caused by non-response in the sample [26, 30].

MI with logistic regression method was utilized to calculate the adjusted prevalences using R-package mice [31, 32]. Ten copies of the analysis data were generated. For each copy, the missing values of gambling participation and problem gambling were imputed using separate imputation models with all socio-demographic factors included in both imputation models before the prevalences were calculated from the imputed data. Finally, the results of ten analysis were combined with the Wald confidence intervals (CIs) of prevalences calculated using R-package miceafter [33]. Differences of crude prevalences against weighted and adjusted prevalences were tested using t-test. The data were analysed using R software version 4.2.2 [34].

## Results

**Fig. 2:**
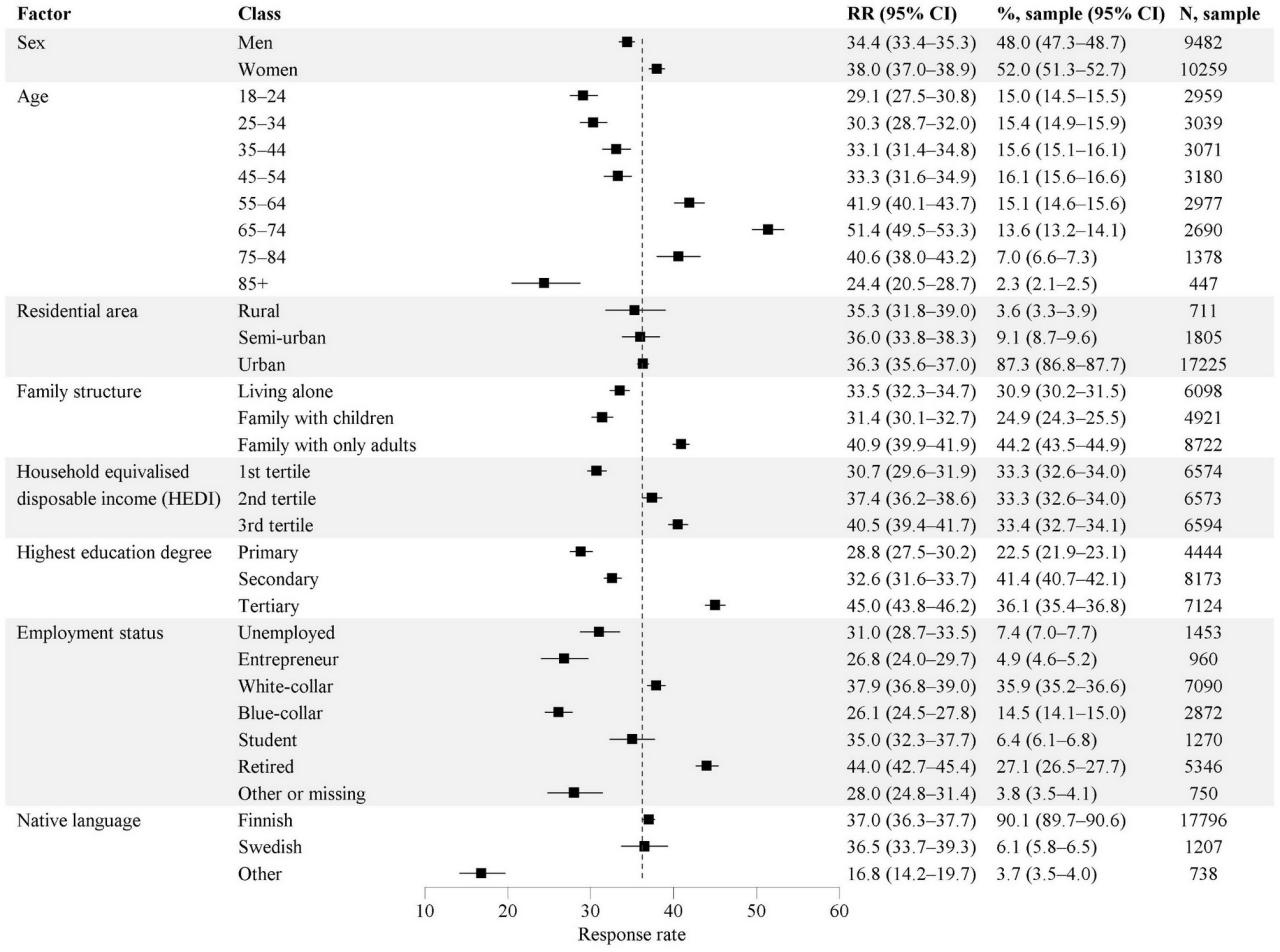
Response rates by socio-demographic factors. Note: dashed line = total response rate of the study sample was 36.2%

Response rates in all subgroups of socio-demographic factors are presented in Fig. 2. Response rates varied from 16.8 to 51.4%. Response rates were higher compared to the overall response rate among women, 55–84-year-olds, those in families with only adults, those in second and third HEDI tertile, those with tertiary education, white-collars, and retired. Response rates were lower compared to the overall response rate among men, 18–54-year-olds, 85-year-olds or older, those living alone or with children, those in the first HEDI tertile, those with primary or secondary education, unemployed, entrepreneurs, blue-collars, those with employment status missing or other, and those with native language other than Finnish or Swedish.

Crude, weighted, and adjusted prevalences of gambling participation are presented in Table 1. There were differences in crude prevalences between subgroups in the following socio-demographic factors: sex, age, highest education degree, employment status, and native language. There was no difference between the crude overall prevalence of gambling participation (81.9% [95% CI 81.0–82.8%]) against either the weighted overall prevalence (83.2% [95% CI 82.3–84.0%]) (p-value 0.09), or the adjusted overall prevalence (82.3% [95% CI 81.6–83.0%]) (p-value 0.49). Weighted and adjusted subgroup-specific prevalences were similar compared to the crude prevalences.

**Table 1:** Crude, weighted, and adjusted prevalences for gambling participation

		Crude prevalence (%)		Weighted prevalence (%)			Adjusted prevalence (%)		
		%	CI	%	CI	*p*-value^1^	%	CI	*p*-value^2^
All		81.9	81.0–82.8	83.2	82.3–84.0	0.09	82.3	81.6–83.0	0.49
Sex	Men	86.2	85.0–87.4	87.3	86.1–88.4	0.28	86.9	85.4–88.2	0.47
	Women	78.3	76.9–79.6	79.4	78.0–80.6	0.29	78.1	76.7–79.4	0.84
Age	18–24	75.6	72.6–78.5	76.4	73.3–79.2	0.74	76.5	74.1–78.8	0.65
	25–34	82.7	80.1–85.1	83.8	81.3–86.0	0.56	83.6	80.8–86.1	0.61
	35–44	85.0	82.6–87.1	85.9	83.6–87.9	0.60	85.5	83.3–87.4	0.75
	45–54	87.5	85.3–89.4	88.0	85.9–89.9	0.73	87.6	85.4–89.4	0.96
	55–64	84.9	82.8–86.8	85.8	83.8–87.7	0.55	85.4	83.2–87.4	0.73
	65–74	80.2	77.9–82.3	81.7	79.5–83.7	0.37	80.7	78.4–82.8	0.73
	75–84	74.8	70.9–78.4	75.2	71.1–78.8	0.90	75.4	71.7–78.6	0.84
	≥ 85	62.2	51.8–71.7	62.4	51.8–71.9	0.99	63.5	54.2–71.9	0.86
Residential area	Rural	81.3	75.7–85.9	82.5	77.0–86.8	0.77	81.5	75.0–86.6	0.98
	Semi-urban	82.7	79.4–85.5	84.2	81.1–86.8	0.53	81.8	79.3–84.2	0.67
	Urban	81.9	80.9–82.8	83.1	82.1–84.0	0.12	82.4	81.6–83.2	0.38
Family structure	Living alone	81.4	79.6–83.1	82.8	81.0–84.4	0.32	81.6	80.1–83.1	0.87
	Family with children	82.0	79.9–83.8	83.2	81.3–85.0	0.41	82.2	80.6–83.7	0.84
	Family with only adults	82.1	80.8–83.4	83.4	82.1–84.6	0.24	82.8	81.6–84.0	0.43
Household equivalised disposable income (HEDI)	1st tertile	80.6	78.7–82.3	81.1	79.3–82.9	0.68	80.2	78.4–81.9	0.76
	2nd tertile	83.8	82.2–85.2	85.1	83.6–86.5	0.27	84.5	83.2–85.8	0.45
	3rd tertile	81.2	79.6–82.7	82.9	81.4–84.3	0.16	82.2	80.6–83.7	0.34
Highest education degree	Primary	83.0	80.8–85.1	83.8	81.6–85.8	0.64	81.8	79.1–84.3	0.46
	Secondary	85.2	83.8–86.5	86.3	84.9–87.6	0.31	85.4	84.1–86.7	0.80
	Tertiary	78.7	77.3–80.1	79.5	78.1–80.9	0.47	79.1	77.4–80.7	0.78
Employment status	Unemployed	86.8	83.3–89.8	87.6	84.1–90.4	0.77	86.5	81.6–90.2	0.87
	Entrepreneur	82.1	76.6–86.5	83.7	78.5–87.7	0.68	83.3	78.5–87.2	0.71
	White-collar	83.9	82.4–85.3	84.5	83.1–85.8	0.59	84.0	82.3–85.5	0.92
	Blue-collar	89.7	87.2–91.7	90.9	88.6–92.7	0.51	89.3	86.3–91.7	0.78
	Student	69.4	64.8–73.6	71.1	66.6–75.2	0.60	68.5	64.0–72.7	0.77
	Retired	78.3	76.6–80.0	79.1	77.3–80.8	0.58	78.1	76.5–79.6	0.84
	Other or missing	83.6	77.7–88.2	84.3	78.6–88.8	0.85	84.3	78.6–88.7	0.85
Native Language	Finnish	82.7	81.7–83.6	83.9	83.0–84.8	0.10	83.3	82.4–84.2	0.30
	Swedish	73.5	69.0–77.5	75.4	71.0–79.3	0.56	74.1	68.3–79.2	0.86
	Other	70.8	61.7–78.6	72.2	63.2–79.7	0.83	71.2	61.6–79.2	0.97

^1^p-values of testing the differences between crude and weighted prevalences using t-test

^2^p-values of testing the differences between crude and adjusted prevalences using t-test

Crude, weighted, and adjusted prevalences of problem gambling are presented in Table 2. The crude overall prevalence of problem gambling was 1.9% (95% CI 1.6–2.3%). There was a difference between the crude and the adjusted overall prevalences (2.8% [95% CI 2.5–3.2%]) (p-value 0.002) while there was no difference between the crude and the weighted overall prevalences (2.2% [95% CI 1.9–2.7%]) (p-value 0.26). The weighted subgroup-specific prevalences were similar compared to the crude prevalences, while the adjusted prevalences for men, urban area, and Finnish as a native language were higher than the corresponding crude prevalences.

**Table 2:** Crude, weighted, and adjusted prevalences for problem gambling

		Crude prevalence (%)		Weighted prevalence (%)			Adjusted prevalence (%)		
		%	CI	%	CI	*p*-value^1^	%	CI	*p*-value^2^
All		1.9	1.6–2.3	2.2	1.9–2.7	0.26	2.8	2.4–3.3	0.002
Sex	Men	3.2	2.7–3.9	3.6	3.0–4.4	0.39	4.3	3.6–5.3	0.03
	Women	0.9	0.6–1.2	0.9	0.7–1.3	0.73	1.4	0.9–1.9	0.08
Age	18–24	2.6	1.7–3.9	3.1	2.0–4.7	0.54	3.5	2.2–5.7	0.27
	25–34	2.1	1.3–3.3	2.5	1.6–3.9	0.56	3.3	1.9–5.6	0.18
	35–44	1.6	0.9–2.6	1.9	1.2–3.2	0.56	2.1	1.3–3.4	0.36
	45–54	2.7	1.8–3.9	2.9	2.0–4.2	0.79	3.6	2.4–5.2	0.26
	55–64	2.1	1.4–3.1	2.3	1.5–3.3	0.80	2.8	1.8–4.4	0.30
	65–74	1.5	0.9–2.3	1.7	1.1–2.6	0.70	1.9	1.2–2.8	0.42
	75–84	0.8	0.2–2.1	0.7	0.2–1.9	0.89	1.1	0.4–3.1	0.63
	≥ 85	2.0	0.4–7.9	1.6	0.4–6.8	0.82	2.3	0.3–16.5	0.75
Residential area	Rural	2.4	1.0–5.5	2.7	1.2–5.9	0.88	3.2	1.4–7.0	0.56
	Semi-urban	2.7	1.6–4.3	3.0	1.8–4.9	0.74	3.9	2.4–6.4	0.25
	Urban	1.9	1.5–2.2	2.1	1.8–2.6	0.29	2.7	2.3–3.1	0.004
Family structure	Living alone	2.6	2.0–3.5	3.1	2.3–4.0	0.47	3.9	2.9–5.2	0.05
	Family with children	1.8	1.2–2.7	2.1	1.5–3.1	0.57	2.5	1.8–3.5	0.18
	Family with only adults	1.6	1.2–2.1	1.8	1.4–2.3	0.56	2.2	1.6–2.9	0.12
Household equivalised disposable income (HEDI)	1st tertile	2.7	2.0–3.5	3.1	2.3–4.0	0.51	3.8	2.7–5.2	0.11
	2nd tertile	2.3	1.7–3.0	2.4	1.8–3.1	0.77	3.1	2.3–4.1	0.11
	3rd tertile	1.1	0.7–1.6	1.4	1.0–2.0	0.38	1.5	1.0–2.3	0.27
Highest education degree	Primary	2.8	2.0–4.0	3.1	2.2–4.3	0.74	4.0	2.8–5.7	0.16
	Secondary	2.8	2.2–3.5	3.1	2.5–3.9	0.52	3.6	2.9–4.5	0.10
	Tertiary	0.9	0.6–1.3	0.8	0.6–1.2	0.83	1.1	0.7–1.6	0.45
Employment status	Unemployed	3.1	1.8–5.3	3.8	2.2–6.3	0.63	4.6	2.6–7.8	0.27
	Entrepreneur	1.2	0.3–3.7	1.2	0.4–3.7	0.99	1.9	0.6–5.9	0.49
	White-collar	0.8	0.5–1.3	0.9	0.6–1.4	0.79	1.1	0.7–1.7	0.32
	Blue-collar	4.7	3.3–6.5	5.1	3.7–7.0	0.74	5.6	3.4–9.2	0.50
	Student	2.3	1.2–4.3	2.7	1.4–5.2	0.68	2.9	1.2–6.8	0.58
	Retired	2.1	1.6–2.8	2.4	1.8–3.2	0.58	2.8	2.1–3.9	0.18
	Other or missing	2.4	0.9–5.9	2.7	1.1–6.6	0.86	4.0	1.8–8.9	0.36
Native Language	Finnish	1.9	1.6–2.3	2.2	1.9–2.7	0.30	2.7	2.2–3.3	0.03
	Swedish	1.2	0.4–2.9	1.5	0.6–3.6	0.72	1.8	0.8–3.9	0.44
	Other	5.0	2.0–11.0	5.2	2.2–11.6	0.96	7.1	2.7–17.4	0.50

^1^p-values of testing the differences between crude and weighted prevalences using t-test

^2^p-values of testing the differences between crude and adjusted prevalences using t-test

## Discussion

Our results indicate that there was no difference between crude and adjusted prevalences of gambling participation. However, the adjusted prevalence of problem gambling was higher (2.8%) compared to the crude prevalence (1.9%). These results are line with the fact that same socio-demographic factors which are associated with problem gambling are also associated with non-response, while the same does not apply for gambling participation [11, 13, 14, 19, 20, 22–24]. The inclusion of non-respondents of these underrepresented subgroups through MI may increase the prevalence of overall problem gambling. For gambling participation, the prevalence has been found to be higher among men, older age groups, higher socio-economic groups, and individuals living in rural areas [11, 13, 14, 19], which are subgroups with relatively high response rates. Therefore, MI of gambling participation has no effect on the prevalence estimate. There was no difference between crude and weighted prevalences. Survey weights were assigned to each respondent using factors sex, age, education degree, region, and urban–rural classification. Usually, survey weights are defined using factors associated with non-response to improve representability of the data. However, weights should be calculated using good predictors of the outcome instead of the non-response [35]. With more sophisticated methods such as MI, the adjustment for non-response can be executed case-by-case, depending on outcome. The development of both weighting and adjusting methods is needed to increase the reliability of results.

Our analyses are based on a combination of online and postal population-based surveys, and the study yielded the response rate of 36.2%. Response rates varied largely, from 16.8 to 51.4%, between different subgroups, confirming that certain sub-population are clearly more difficult to recruit than others. Such sub-groups included men, 18–54-year-olds, 85-year-olds or older, those living alone or with children, those in the first HEDI tertile, those with primary or secondary education, unemployed, entrepreneurs, blue-collars, those with employment status missing or other, and those with native language other than Finnish or Swedish. Correspondingly, response rates were higher than the overall response rate among women, 55–84-year-olds, those in families with only adults, those in second and third HEDI tertile, those with tertiary education, white-collars, and retired. These results are in line with previous research where non-response rate is usually higher among men, the young ones, the single ones and in lower socio-economic groups [6, 18, 21]. The response rate of our study was higher than the international average of 29.0% for online and postal surveys [5]. Moreover, our response rate is comparable to the response rates in recent Nordic population-based studies: 26.0% in Norway, 28.5% in Sweden, 36.9% in Finland, 37.6% in Denmark, and 42.0% in telephone interview in Iceland [11, 13, 14, 19, 20]. These studies had rather large variation in both methodology and response rates, even in data set collected in rather similar time points and cultural contexts.

These results reinforce the growing need for targeting socio-demographic subgroups with specific data collection measures. For young men, which are one of the most challenging subgroups, possibility to answer to short version first, possibility to select interesting themes from questionnaires, and increased comprehensibility and plain language have been identified as factors as preliminary ideas for increasing response rate [36]. Generally, response rates could be increased by introducing mixed-mode surveys, where potential respondents are contacted with survey mode maximizing their probability to respond. As a simple example, older age groups are more likely to respond to paper questionnaire than younger age groups [37]. Non-response analysis can be used for profiling the individuals in the study sample and designing the data collection based on each profile. In this study, the response rate was relatively high until 85-year-old respondents. Response rates for older age groups may remain high in the future, since the population is aging, and older age groups have better functioning and are more active than their predecessors [38]. Therefore, the population-based surveys should be conducted without upper age limit for study sample to prevent the exclusion of increasingly large part of the population and thus jeopardizing the representativeness of a study sample.

To our knowledge, this study is the first to analyse both non-response of a population study and the effect of non-response with gambling-related outcomes. Similar types of sophisticated analyses have been performed with several other health surveys focusing on smoking [39], alcohol and other drug use [40], and primary health care utilization [25]. In this paper, MI was utilized to calculate the adjusted prevalences of gambling participation and problem gambling by register-based variables: sex, age, residential area, family structure, HEDI, highest education degree, employment status, and native language. Obtaining additional information from administrative registers enables more thorough analysis of the structure of non-response and exploiting MI provides prevalence estimates when controlling for possible non-response bias. Overall, previous gambling-related non-response analyses are mainly based on reporting the subgroup-specific response rates [6, 13, 14, 21].

Despite the development of statistical methods reducing non-response bias, maintaining as high as possible response rates is essential. If the response rates continue to decrease, at some point statistical methods are unable correct the bias, although the decrease of response rate does not necessarily mean the increase in non-response bias [41, 42]. Personalized contact with potential respondents, using a more interesting topic, shorter and well-formatted questionnaires, and incentives are factors associated with higher response rate [43, 44]. Also, concerns about privacy in online surveys [45] must be addressed in a digitalizing society.

Individual-level data from administrative registers offers an excellent opportunity to exploit more sophisticated statistical methods in the analysis of non-response. Countries with register data should be encouraged to invest extra effort to further exploit register data. The incorporation of health information to non-response analysis would probably bring added value to non-response analysis, since there is association between health status and non-response [46–49].

### Strengths and limitations

Our analyses are based on a combination of online and postal population-based survey with a response rate of 36.2%. In the basic report [26] and in other publications [50–54], the response rate was reported as 36.1%. This small difference in response rates is explained by the exclusion of 192 individuals, who had missing register data. In addition, those answering the paper questionnaires tended to be older than those choosing the online survey [26]. Furthermore, gambling participation, particularly online gambling, as well as at-risk gambling and problem gambling were more common among those who participated using the online survey compared with those using the postal version [26]. The results of the latest Finnish gambling population study comparing different survey modes indicates that respondents and their gambling behaviour differs between computer-assisted telephone interview (CATI), and online and postal survey [19]. The prevalence of online gambling was higher among online and postal respondents compared to CATI respondents, while the prevalence of only land-based gambling was higher among CATI respondents. Also, the prevalence of low-risk gambling was higher in online and postal survey than in CATI.

Furthermore, it is known that socially desirable responding cause response bias affecting the accuracy of self-assessments. This notion is important while studying sensitive topics, such as problem gambling. In the context of addictions research, controlling the effect of socially desirable responding on self-reported measures should be considered as a method to help reduce error and improve validity [55]. Our analyses are based on data collected in 2017 and there has been changes in Finnish gambling environment since due to policy changes as well as transnational gambling trends [56]. For instance, the prevalence of gambling non-monopoly online games has increased [19]. Thus, our conclusions might be outdated, and the analysis need to be repeated with newer data to address this.

## Conclusions

Non-response had an effect of problem gambling prevalence in the Finnish Gambling Harms Survey 2016. The presence of non-response bias should be checked when analysing population surveys. Using administrative register data enables unique opportunities to increase the reliability of the results and to adjust the estimates for non-response.

## Electronic supplementary material

Below is the link to the electronic supplementary material.

Supplementary Material 1

## Data Availability

The Finnish Gambling Harms dataset is available from the Finnish Social Science Data Archive [57]. The register-based data was available in FIONA, the protected environment of Statistics Finland [58]. The latter required a separate contract with THL and Statistics. The results were transferred from FIONA to the authors through a screening process.

## Abbreviations

CATI: Computer-assisted telephone interview
CI: Confidence interval
Fohm: Folkhälsomyndigheten
HEDI: Household equivalised disposable income
MI: Multiple imputation
OECD: Organisation for Economic Co-operation and Development
PPGM: Problem and Pathological Gambling Measure
SCB: Statistics Sweden
THL: Finnish Institute for Health and Welfare
