# Supplementary material for: Using administrative register data for adjusting non-response bias in the finnish gambling harms survey
Source: BMC Public Health. 2025 May 16;25:1807. doi: 10.1186/s12889-025-23016-4 (PMC12083128; doi:10.1186/s12889-025-23016-4)
Supplement: Supplementary file 1 — Supplementary Material 1 [file 12889_2025_23016_MOESM1_ESM.docx]

**Using administrative register data for adjusting non-response bias in the Finnish Gambling Harms Survey (Supplementary material)**

**Supplementary figure 1. Response rate by age in the Finnish Gambling Harms Survey 2016**


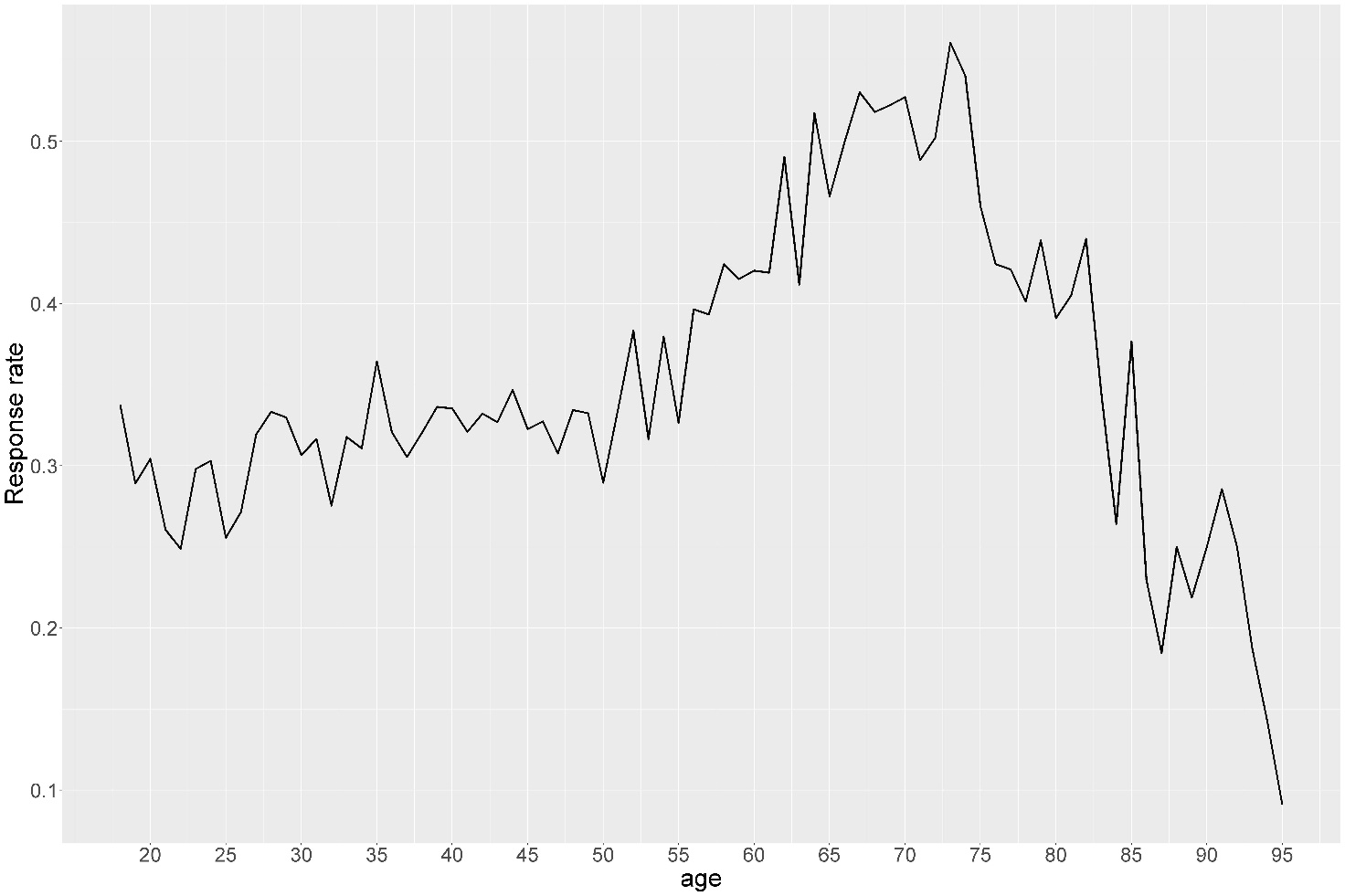


Note: Individuals older than 95 years excluded from the figure due to small sample size.

R-code for calculating prevalence and confidence interval with multiple imputation

library(mice)

library(miceafter)

mi.vars <- c(”outcome”,”f1”,”f2”,”f3”,”f4”,”f5”,”f6”,”f7”,”f8”)

dat.mi <- subset(data, select=mi.vars)

pred.m <- matrix(0, ncol(dat.mi), nrow(dat.mi))

dimnames(pred.m) <- list(colnames(dat.mi), colnames(dat.mi))

pred.m[”outcome”,] <- 1

diag(pred.m) <- 0

mi.outcome <- mice(dat.mi,

m=10,

maxit=10,

method=c(”logreg”),

predictorMatrix=pred.m,

print=FALSE,

seed=53463)

#Prevalence:

pool(with(mi.outcome, expr=lm(as.character(outcome)~1))) #result

#Confidence interval:

ci.outcome <- mids2milist(mi.outcome, keep=TRUE)

ci.outcome <- with(mids2milist(mi.outcome, keep=TRUE),

expr=prop_wald(outcome~1))

pool_wald(ci.outcome) #result
